# Diagnostic Performance of a Deep Learning-Based Tool for the Detection and Staging of Rectal Cancers on Endoscopic Ultrasound: Prospective Study

**DOI:** 10.3390/diagnostics16081161

**Published:** 2026-04-14

**Authors:** Amedeo Montale, Marco Valvano, Andrea Ghezzi, Marco Magliocco, Nicola Panvini, Mariangela Rutigliani, Massimo Oppezzi, Lucio Molini, Francesco Paparo

**Affiliations:** 1 Gastroenterology Unit, E.O. Ospedali Galliera, Mura delle Cappuccine 14, 16128 Genoa, Italy; amedeo.montale@galliera.it (A.M.); andrea.ghezzi@galliera.it (A.G.); massimo.oppezzi@galliera.it (M.O.); 2 Department of Experimental Medicine, University of Genoa, 16126 Genoa, Italy; marco.magliocco@camelotbio.com; 3 Diagnostic Imaging Unit, E.O. Ospedali Galliera, Mura delle Cappuccine 14, 16128 Genoa, Italy; nicola.panvini@galliera.it (N.P.); lucio.molini@galliera.it (L.M.); francesco.paparo@galliera.it (F.P.); 4 Pathology Unit, E.O. Ospedali Galliera, Mura delle Cappuccine 14, 16128 Genoa, Italy; mariangela.rutigliani@galliera.it

**Keywords:** rectal endoscopic ultrasound, transrectal ultrasound, rectal cancer, deep learning, artificial intelligence, computer-aided detection

## Abstract

**Background**: Loco-regional staging of rectal cancer relies on MRI and rectal EUS (R-EUS). In situ and T1 tumors may be candidates for endoscopic resection, and R-EUS enables reliable differentiation among early-stage tumors (Tis, T1, T2), a distinction that MRI cannot consistently provide. This prospective pilot study aimed to evaluate the predictive performance of a deep learning (DL) tool for detecting and staging rectal tumors on R-EUS. **Methods**: The DL tool uses a convolutional neural network for image segmentation and classification. Performance in lesion segmentation was assessed using the Dice Similarity Coefficient (DSC) and F1-score. The model was first evaluated for its ability to differentiate in situ and T1 tumors from T2/T3 lesions, and then for distinguishing Tis from other stages (T1, T2, T3). **Results**: Fifty patients were enrolled. The median DSC for tumor segmentation was 0.65 (IQR 0.17). Tumor detection showed an accuracy of 0.77, precision of 0.85, recall of 0.77, and F1-score of 0.81. In distinguishing Tis/T1 from T2/T3 tumors, the model achieved an accuracy of 0.64, precision of 0.88, recall of 0.64, and F1-score of 0.74. For distinguishing Tis from T1, T2, and T3 lesions, the accuracy was 0.80, precision 0.83, recall 0.89, and F1-score 0.86. Mesorectal lymph node segmentation showed a median DSC of 0.62 (IQR 0.17). **Conclusions**: The DL tool shows promise for aiding operators in identifying rectal lesions suitable for endoscopic resection. The semi-supervised training approach reduces manual segmentation burden and achieves performance comparable to expert physicians.

## 1. Introduction

Colorectal cancer is the third most common malignancy worldwide. Loco-regional staging is primarily performed using magnetic resonance imaging (MRI) and ultrasound techniques with endocavitary transducers—mainly transrectal ultrasound (TRUS) and rectal EUS (R-EUS)—and is essential for therapeutic decision-making. It is based on the assessment of tumor depth (T) and regional lymph node involvement (N) according to the TNM classification system [1,2]. Rectal MRI does not reliably distinguish early T categories (Tis, T1, T2), except in selected patients with T1 tumors in whom a preserved submucosal layer (hyperintense signal) beneath the lesion can be identified [3,4].

Therefore, TRUS or R-EUS is recommended in these cases because of their superior spatial resolution and diagnostic performance [5,6,7]. Clinically, however, both TRUS and R-EUS are operator-dependent techniques with a relatively long learning curve, and substantial discrepancies may occur between examinations performed by experienced and novice physicians [8,9]. Computer-aided detection (CAD) tools and artificial intelligence (AI) deep learning (DL) models have been successfully applied in rectal cancer, especially for enhancing radiomic analyses on rectal MRI [10,11,12,13].

More recently, AI- and DL-based tools have also been proposed for the detection and staging of rectal cancer on TRUS images, yielding promising results [14,15,16]. Carter et al. utilized fine-tuned DL architectures to analyze a dataset of 294 TRUS images acquired with a radial transrectal transducer [15].

Their CAD platform achieved an area under the curve (AUC) of 0.85 for rectal cancer diagnosis, employing a self-supervised learning approach that allows for model training on unlabeled data [17]. Liu et al. recently developed a CAD tool based on a DL model applied to 3D-TRUS images, demonstrating strong performance in T staging and improving both the diagnostic confidence of less-experienced operators and inter-observer consistency [16]. To date, only one study has specifically addressed DL-assisted segmentation and analysis of R-EUS images of rectal tumors [9]. Van den Noort et al. developed an automated segmentation tool for R-EUS examinations performed with a linear probe, aiming to standardize ultrasound interpretation and improve the detection of rectal polyps and early rectal cancers [9]. They reported a median Dice similarity index (DSC) of 0.82 for rectal tumor segmentation, indicating good agreement between manual and DL-based segmentations. As highlighted by Podda et al., further research is necessary to improve automatic segmentation of TRUS and R-EUS images, which are highly heterogeneous due to intrinsic tumor characteristics and the frequent presence of artifacts [8].

Given the promising results of the few earlier studies, our primary objective was to investigate the diagnostic potential of a novel DL-assisted tool trained with a semi-supervised learning approach for the detection and staging of rectal cancer on R-EUS examinations performed with a radial transducer. A preliminary evaluation of the model’s performance in segmenting mesorectal lymph nodes was also conducted.

## 2. Materials and Methods

This single-center prospective pilot study included 50 consecutive patients with rectal tumors who were referred to the Gastroenterology Unit of our Institution to undergo diagnostic R-EUS following a positive colonoscopy. The study received approval from the Ethics Committee of N.CET-Liguria (289/2023-DB ID 13225; 4 December 2023). Written informed consent was obtained from all patients prior to enrollment, which took place between May 2024 and December 2024.

Most included patients had undergone colonoscopy due to symptoms suggestive of colorectal disease—such as rectal bleeding, abdominal pain, or weight loss—or for colorectal cancer screening.

### 2.1. Inclusion and Exclusion Criteria

- age ≥ 18 years;
- provision of written informed consent;
- Endoscopic detection of rectal lesions with high-grade dysplasia or a neoplastic superficial pattern of grade 2B/3 according to the JNET classification [18];
- Patients scheduled for endoscopic mucosectomy or with adenomatous rectal lesions confirmed by histopathology were excluded.

### 2.2. Study Procedures

All patients with T stage >1 underwent preoperative MRI performed on a 1.5T system (SIGNA Explorer 1.5T, GE Medical Systems, Little Chalfont, UK) following a standardized protocol for rectal cancer staging [1]. The R-EUS image dataset was processed in anonymized form by Camelot Biomedical (Camelot Biomedical Systems S.r.l., Genoa, Italy) in full compliance with ethical guidelines and the European Union’s General Data Protection Regulation (GDPR, Regulation [EU] 2016/679).

*Rectal endoscopic ultrasound*

R-EUS examinations were performed by two physicians (M.A. and V.M.) with expertise in colorectal endoscopy and R-EUS. The Olympus GF-UE160 endoscope (Tokyo, Japan), equipped with a 360° radial transducer operating at 5–20 MHz, was used for all procedures. Patients were instructed to perform two cleansing enemas prior to the examination. The rectum was then distended with a 250–300 mL water enema, and the endoscope was advanced to the distal sigmoid colon. R-EUS images were acquired as video datasets, and the operators documented the relevant morphological and dimensional characteristics of the rectal lesions. T and N staging were performed according to the TNM classification system [2].

Ultrasound technical parameters, including gain, frequency, and time-gain compensation, were adjusted at the operator’s discretion to obtain optimal image quality for each examination. To minimize air-related artifacts, both air suction and a water-inflated balloon mounted on the ultrasound transducer were utilized.

*Deep Learning model building*

**Figure 1:**
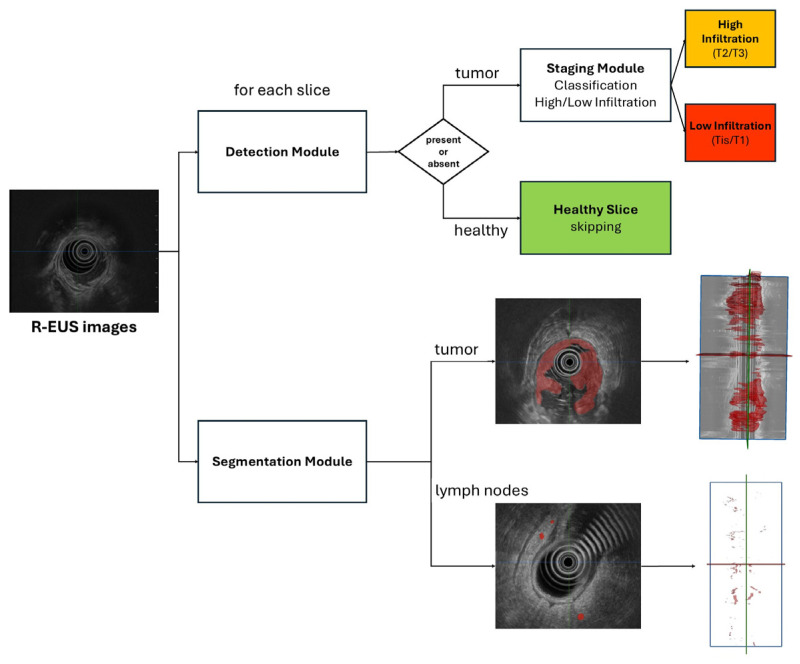
The flowchart of DL model building. For the segmentation pipeline, a Feature Pyramid Network (FPN) was employed. T stages were dichotomized. In the first analysis, Tis and T1 stages were considered absent/low infiltration, whereas T2 and T3 stages were classified as higher infiltration. In the second analysis, Tis was considered the absence of infiltration, while T1, T2, and T3 stages were classified as higher infiltration.

The main objective of our CNN-based tool was the preoperative segmentation, detection, and staging of rectal tumors on R-EUS scans using a DL model. Two separate DL models were developed: one for morphological segmentation of rectal lesions and the other for tumor staging (Figure 1). All R-EUS videos were anonymized and subsequently converted into sets of image frames using a photogram fractionation/division tool to facilitate further processing. The resulting R-EUS image sets were manually segmented by the same two physicians who performed the examinations (M.A. and V.M.), using the Medical Imaging Interaction Toolkit [19]. Both operators were blinded to all demographic, clinical, and histopathological information. They delineated cancerous tissue within the rectal wall layers—including tumor extensions into the mesorectum—to create annotations for training the segmentation model.

To optimize the annotation workload and maximize the information extracted from each examination, only a representative subset of frames was selected for manual segmentation, focusing on images that best depicted the relevant anatomical and pathological features. Specifically, the operators first excluded repeated or highly similar frames from each R-EUS examination to avoid redundancy. Subsequently, particular attention was paid to selecting frames that best captured the areas of maximal infiltration of the rectal wall.

For training the classification pipeline, the same operators assigned T and N stages to each R-EUS frame containing pathological tissue according to the TNM classification system, where T0 indicates no evidence of primary tumor and higher T categories reflect deeper invasion into rectal wall layers and surrounding perirectal structures:

- Tis (carcinoma in situ): intraepithelial tumor or invasion of the lamina propria;
- T1: tumor invasion of the submucosa;
- T2: tumor invasion of the muscularis propria;
- T3: tumor infiltration through the muscularis propria into mesorectal fat without reaching the mesorectal fascia or adjacent organs;
- T4: tumor penetration of the visceral peritoneum or direct invasion/adhesion to adjacent organs or structures.

Following Liu et al. [16], after lesion segmentation, frames that did not directly display cancerous tissue were removed to reduce computational load and focus the network’s attention on the most informative tumor-containing images. After filtering out non-informative frames, the final R-EUS dataset consisted of 5794 annotated tumor slices for the segmentation model and 23,288 annotated images for the classification model. Of these, 14,844 frames contained tumor tissue (Tis: 4650; T1: 2598; T2: 3960; T3: 3636), while 8444 were healthy frames showing no tumor involvement of the rectal wall. Additionally, because 12 of the 50 patients presented mesorectal lymphadenopathy (N+), a total of 1322 slices were annotated to assess the DL algorithm’s ability to detect lymph nodes.

*Deep Learning pipeline*

The deep learning pipeline was implemented using a ResNet34 backbone pretrained on ImageNet. Previous research has demonstrated that employing a pretrained backbone enables the model to leverage feature representations learned from large natural image datasets, providing a robust initialization for training. This strategy is particularly advantageous when the available dataset is limited, as it reduces the risk of overfitting and improves the model’s ability to generalize to new cases, ultimately enhancing segmentation performance [20].

All R-EUS images underwent a standardized preprocessing pipeline to ensure appropriate formatting and scaling for subsequent analysis by the neural network. Specifically, pixel values were normalized to the [0, 1] range to accelerate and stabilize training, and all images were rescaled to 256 × 256 pixels to maintain dimensional consistency. The model was trained using the Adam optimizer with an initial learning rate of 0.001. A Reduce-on-Plateau learning rate scheduling strategy was adopted, whereby the learning rate was decreased by a factor of 0.7 when the validation performance (Dice score) plateaued. The training process was carried out for a maximum of 500 epochs, with an early stopping criterion implemented using a patience of 20 epochs, monitoring the Dice score on the validation set. Training was performed with a batch size of 4, using input images with a spatial resolution of 256 × 256 pixels. All experiments were conducted on a workstation equipped with an NVIDIA RTX 2080 Ti GPU with 11 GB of VRAM, an Intel Core i9-9900K CPU running at 3.60 GHz, and 32 GB of RAM.

Model training employed cross-validation. A 20-fold cross-validation strategy was used for both tumor segmentation and classification. At each iteration, one subset of data served as the test set, while the remaining subsets were divided into training (85%) and validation (15%) sets. Final performance metrics were computed by aggregating results from all test folds. For lymph node segmentation, a 14-fold cross-validation approach was adopted due to the smaller number of available R-EUS frames.

*Tumor and lymph node segmentation model*

For the segmentation pipeline, a Feature Pyramid Network (FPN) architecture was employed [21], allowing for the extraction of multiscale features, an essential capability given the considerable variability in tumor shape and size. The network was trained using Dice Loss, and training progress was monitored with an early-stopping criterion based on the DSC calculated on the validation set. Following model training, segmentation performance was evaluated using the DSC, a widely adopted metric for quantifying the overlap between predicted and ground-truth segmentations.

*Tumor Classification model*

The classification model was tasked with staging rectal tumors according to the TNM classification system. The pipeline first employed a classification network to determine whether each R-EUS frame displayed tumor tissue within the rectal wall. For frames classified as positive, a second classification model was applied to determine tumor stage. Two separate analyses were performed by dichotomizing the four T categories into two clinically relevant classes: tumors with absent or low infiltration and those with higher levels of local invasion (T2 and T3). In the first analysis, Tis and T1 were categorized as absent/low infiltration, whereas T2 and T3 were classified as higher infiltration. In the second analysis, Tis was considered the absence of infiltration, while T1, T2, and T3 were grouped as higher infiltration.

To reduce uncertainty associated with human annotation, ultrasound frames showing transitions between different depths of mural invasion were excluded from the training dataset. Model training was performed using cross-entropy loss with class weighting to address class imbalance. Performance was assessed using accuracy, recall, precision, and the F1-score—the harmonic mean of precision and recall, providing a balanced measure of both metrics. Confusion matrices were generated to provide a comprehensive visualization of model performance and classification errors.

Because determination of the final T stage depends on a relatively small number of key frames depicting the maximum invasion depth, we applied a 15% threshold to improve case-level T-stage prediction. Specifically, if ≥15% of the key frames for a given patient were classified as showing T2 invasion, the model assigned a T2 stage to that case.

Model performance in T staging (compared with the reference standard) was evaluated using area under the receiver operating characteristic curve (AUC–ROC) analysis. Agreement between the DL-based model and human operators was assessed using weighted Cohen’s kappa, and a rank correlation coefficient was calculated to compare the staging assigned by the CNN-based tool with the reference standard.

*Lymph node Classification model*

The segmentation pipeline was further extended to the detection of mesorectal lymph nodes. Due to the limited size of the dataset, a data augmentation strategy was implemented by including blood vessels in the segmentation task, providing an additional 4269 segmented/annotated slices. Blood vessels share morphological similarities with lymph nodes and can be easily misclassified in 2D segmentation, as 3D spatial information is not available. Accurate discrimination between lymph nodes and vessels can be achieved by considering structural roundness and the extent of their appearance across consecutive slices. Lymph nodes, which are roughly spherical, typically appear in only a few consecutive slices, whereas mesorectal vessels are tubular structures extending across many slices—providing a key criterion for differentiation.

DL model performance was first evaluated by calculating the overall DSC for both mesorectal lymph nodes and vessels. Additionally, a lymph node-specific assessment was performed by defining a restricted region of interest around each mesorectal lymph node using the ground-truth segmentation and computing the DSC specifically within these regions.

*Standard of reference*

All clinical cases were discussed in a multidisciplinary team to determine the most appropriate therapeutic strategy for each patient, taking into account the available medical records, including endoscopy, histopathological analysis of endoscopic biopsies, R-EUS, and MRI examinations. Comorbidities were also considered in the decision-making process. Pathological analysis of endoscopically resected or surgical specimens was used as the reference standard whenever available.

Twenty-one patients, including 16 with Tis and 5 with T1 rectal tumors, were deemed suitable for endoscopic resection via endoscopic submucosal dissection (ESD). Twenty-four patients underwent upfront surgery, comprising 13 T2N0, 4 T2N+, 1 T3N0, and 6 T3N+ cases. Among the remaining five patients with T3 rectal tumors, four (1 T3N+ and 3 T3N0) underwent surgery following neoadjuvant chemoradiation therapy, while one patient (T3N+) received radiotherapy alone. All patients treated with neoadjuvant chemoradiation therapy underwent preoperative rectal MRI, which was considered the reference standard for staging.

## 3. Results

Between May 2024 and December 2024, a total of 77 patients underwent R-EUS in our Gastroenterology Unit; of these, 50 patients met the inclusion criteria and were prospectively enrolled in the study. Demographic, clinical, and pathological characteristics of the study population are summarized in Table 1. According to the reference standard, the distribution of T and N categories was as follows: Tis 16/50, T1 5/50, T2N0 13/50, T2N+ 4/50, T3N0 4/50, and T3N+ 8/50. No T4 tumors were identified in this cohort.

### 3.1. Tumor Segmentation

**Figure 2:**
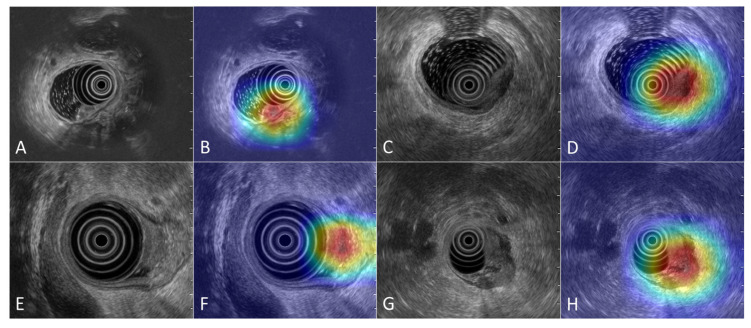
R-EUS images of rectal tumors at different stages, with the corresponding regions of interest (ROIs) delineated within the rectal wall by the DL tool. In R-EUS images, the rectal tumor is displayed as a hypoechoic mass that disrupts the normal echo-layer pattern of the rectal wall. R-EUS images (**A**,**B**) show a case of Tis, (**C**,**D**) a T1 tumor, (**E**,**F**) a T2 tumor, and (**G**,**H**) a T3 tumor.

The segmentation analysis of the test set yielded a median DSC of 0.65, with an interquartile range of 0.17. Examples of tumor segmentation across different T stages performed by the DL-based tool are shown in Figure 2.

### 3.2. Lymph Node Segmentation

**Figure 3:**
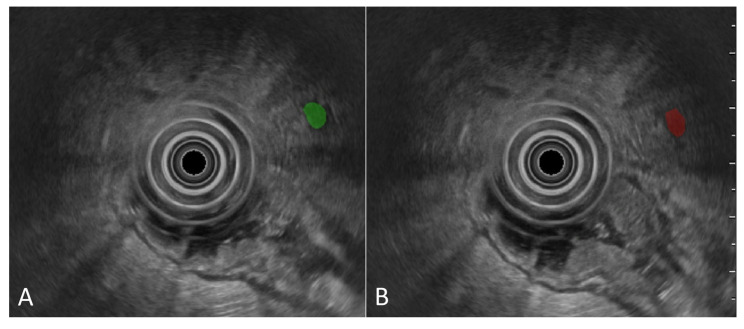
Example of mesorectal lymph node segmentation on R-EUS image frames. Image (**A**) displays in green the ground truth (manual annotation by human operators); image (**B**) shows in red the prediction provided by the DL tool.

The segmentation of mesorectal lymph nodes and blood vessels yielded a median DSC of 0.62 (IQR 0.17). When the model’s performance was assessed by restricting the analysis to regions surrounding the mesorectal lymph nodes—thereby excluding potentially confounding structures such as blood vessels—the DSC increased to 0.79 (IQR 0.21). An example of mesorectal lymph node segmentation performed by the DL-based tool is shown in Figure 3.

### 3.3. Diagnostic Performance in Tumor Detection and T Staging

**Figure 4:**
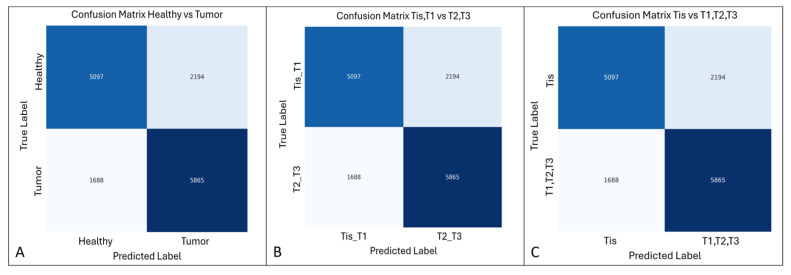
(**A**) Confusion matrix for tumor detection (healthy/tumor classification) obtained by aggregating the 20 test sets from cross-validation. (**B**) Confusion matrix for tumor staging classification in the dichotomization Tis, T1 vs. T2, T3. (**C**) Confusion matrix for tumor staging classification in the dichotomization Tis vs. T1, T2, T3.

The classification network for tumor detection achieved an accuracy of 0.77, precision of 0.85, and F1-score of 0.81. The corresponding confusion matrix, aggregated across all cross-validation test sets, is shown in Figure 4A.

For the test dataset, the DL model’s performance in discriminating Tis/T1 versus T2/T3 stages was as follows: accuracy 0.74, precision 0.73, recall 0.77, and F1-score 0.75 (Table 2). The confusion matrix for this first dichotomization is presented in Figure 4B. When discriminating Tis versus T1/T2/T3 stages, the model achieved an accuracy of 0.80, a precision of 0.83, a recall of 0.89, and an F1-score of 0.86 (Table 3), with the corresponding confusion matrix shown in Figure 4C.

The inter-rater agreement between human operators and the DL model yielded a weighted Cohen’s Kappa of 0.88 (standard error 0.04; 95% CI: 0.80–0.97). The rank correlation coefficient comparing CNN-based staging with the reference standard was 0.93 (95% CI: 0.88–0.96).

In the testing cohort, the DL model demonstrated strong diagnostic performance across individual T stages. Diagnostic accuracy for Tis, T1, T2, and T3 tumors was 89.8% (AUC 0.85; 95% CI: 0.72–0.94), 85.7% (AUC 0.66; 95% CI: 0.51–0.79), 89.8% (AUC 0.91; 95% CI: 0.79–0.97), and 93.9% (AUC 0.90; 95% CI: 0.78–0.97), respectively, with the highest accuracy observed for T3 tumors. By aggregating Tis and T1 stages, the model achieved an overall diagnostic accuracy of 95.9% (AUC 0.95; 95% CI: 0.85–0.99) (Table 4).

## 4. Discussion

With the improvement of screening strategies, the detection of rectal polyps and early rectal cancers has significantly increased [9]. T stage remains one of the key prognostic factors used in clinical guidelines to determine the most appropriate therapeutic approach in rectal tumors. In situ lesions (Tis) and tumors confined to the submucosa (T1) can be managed with endoscopic resection techniques, primarily endoscopic submucosal dissection. Lesions that extend into, but not beyond, the muscularis propria (T2) are clinically considered early-stage tumors and are generally treated with upfront surgery alone [1,2]. Once the muscularis propria is infiltrated, the risk of lymph node metastasis rises to 25–30% [22,23,24].

Distinguishing early T stages (Tis and T1) from more advanced disease (T2, T3, T4) remains a major clinical and diagnostic challenge. In this regard, R-EUS and TRUS provide real-time, high-resolution visualization of rectal wall layers and are particularly useful for differentiating among clinical Tis, T1, and T2 stages. Conversely, rectal MRI does not reliably distinguish these early categories and may lead to overstaging [25]. However, both TRUS and R-EUS are highly operator-dependent techniques, and their diagnostic accuracy outside high-volume tertiary centers is often lower. In addition, the learning curve can vary considerably among operators and is influenced by the number of examinations performed at each institution [8,15].

The integration of AI, CAD, and radiomics into rectal cancer diagnosis and risk stratification has shown promising results, with the potential to enhance diagnostic accuracy and improve staging precision [26,27,28,29]. Convolutional neural networks, a class of deep learning models designed to process medical images, have been increasingly applied to automated T staging of rectal cancer on MRI [13]. These models can also be combined with self-supervised learning, an approach that enables training on unlabeled data [8,9]. To date, the development of DL-based CAD tools has primarily focused on rectal MRI, which remains the reference imaging technique for accurate assessment of T3 tumors and for mesorectal lymph node evaluation [11,12,13,27,28,29]. A recently developed CNN-based model using MRI data outperformed human operators in preoperative T staging of rectal cancer (AUC 0.854 vs. 0.678) [13].

Recently, several operator-supporting DL-based systems for the detection of polyps and rectal cancers have been proposed to improve the accuracy of TRUS and R-EUS examinations performed by less experienced clinicians, thereby potentially reducing the risk of misdiagnosis [8,9,14,15,16,30]. Extracting morphological and volumetric information from TRUS and R-EUS images can indeed be challenging for operators with limited experience or in centers with low procedural volumes. A DL model developed by Chang et al. to distinguish rectal cancer from normal rectal wall on TRUS images achieved a validation accuracy of 90.5% and an AUC of 0.945 [14]. In the study by Liu et al., the CAD tool showed its best performance in identifying T1 tumors (accuracy 91.7%, AUC 0.85), while achieving lower performance for T2 and T3 lesions [16]. Van den Noort et al. made an initial attempt to develop an automatic segmentation tool to facilitate the interpretation of R-EUS images of rectal polyps and early rectal cancers obtained with a linear probe; their CNN-based model successfully segmented rectal wall layers and lesions in 80.4% of cases [9].

To our knowledge, this is the first study focusing on the development of a CNN DL model for the detection of rectal cancer on R-EUS examinations performed with a radial transducer.

In our study, the DSC value for tumor segmentation was 0.65, notably lower than the 0.82 DSC reported by van den Noort et al. [9]. Similarly to van den Noort et al., we selected only a representative subset of frames for manual segmentation to optimize the annotation workload; however, our study included a substantially larger number of ultrasound images. Therefore, it is unlikely that the comparatively lower DSC observed in our results is solely attributable to an insufficient number of annotated images. A more plausible explanation for the discrepancy in DSC values may relate to other factors, including the greater morphological and structural heterogeneity of rectal lesions in our cohort. In addition, radial EUS probes provide a more panoramic view than linear probes. While this allows for a broader circumferential assessment of the rectal wall, the representation of the wall layers may be less uniform, increasing the complexity of the segmentation task for the DL model and potentially resulting in lower DSC values compared with the more focused sectorial images obtained with linear probes. Importantly, despite the relatively modest overall DSC, the model’s classification performance remained satisfactory.

Our results demonstrate that the proposed DL model achieved good diagnostic performance in the assessment of rectal tumor T stage, with accuracies ranging from 85.7% to 93.9% across individual stages and reaching 95.9% when early lesions (Tis–T1) were grouped together. The model showed particularly strong performance for T2 and T3 tumors, with accuracies of 89.8% and 93.9% and corresponding AUC values of 0.91 and 0.90, respectively. When compared with conventional staging techniques, it is important to consider the stage-specific strengths of currently available imaging modalities. Previous studies on R-EUS have reported diagnostic accuracies of approximately 80–90% for T1 tumors and 70–85% for T2 tumors, highlighting its particular value in the identification of superficial lesions and early wall invasion [6,7]. However, its performance tends to decrease in more advanced tumors, especially in the presence of bulky or stenotic lesions that may limit complete endoluminal assessment [7]. Conversely, pelvic MRI is currently considered the reference modality for the evaluation of locally advanced rectal cancer, primarily because of its ability to assess extramural tumor extension and its relationship with the mesorectal fascia [1,4]. Reported diagnostic accuracies for MRI are generally lower for early stages, with values of approximately 60–75% for T1–T2 tumors, but increase for more advanced disease, reaching 75–90% accuracy for T3–T4 tumors, where evaluation of mesorectal invasion and the circumferential resection margin becomes critical for treatment planning [1,4]. In this context, the diagnostic performance observed in our study suggests that the DL approach may achieve a level of accuracy comparable to, and in some scenarios potentially exceeding, that of these conventional techniques. Notably, the model demonstrated excellent discrimination between superficial lesions and more advanced tumors, with an AUC of 0.95 for the combined Tis–T1 category, which is clinically relevant for identifying patients who may benefit from organ-preserving strategies.

In our work, we addressed the technical issues inherent to the processing and manipulation of ultrasound images. R-EUS examinations were performed using a 2D 360° radial probe, which provides a panoramic circumferential view of the rectal wall but lacks spatial information along the *z*-axis, an acknowledged limitation when ultrasound scans are used for fusion imaging or for segmentation tasks [8].

The promising results obtained in our study in regard to lesion segmentation suggest that segmentation-derived information could be used to obtain quantitative measures of tumor extent, provided that pixel size is known. This would allow for the standardization of geometric parameters, making the assessment more objective and comparable across patients and medical centers.

Our study has various limitations, most notably the limited size of our study cohort, which is consistent with the nature of a pilot study. In addition, the DL model was validated only using cross-validation on the same dataset and has not yet been tested on an independent external cohort. Nevertheless, the promising performance of the DL model in staging rectal tumors on R-EUS images encourages us to plan future multicenter studies to externally validate the model on larger and more heterogeneous datasets, including patients from multiple institutions. Another relevant limitation is the inclusion of patients who underwent neoadjuvant chemoradiation therapy. In these cases, preoperative MRI, performed prior to neoadjuvant treatment and in close temporal proximity to R-EUS, may serve as a reasonable surrogate for the pathological assessment of surgical specimens. However, the number of patients who received neoadjuvant chemoradiation therapy in our cohort was limited, and we believe that their inclusion did not substantially influence the overall study results [16].

The semi-supervised training on unannotated data enabled substantial savings in time and resources, and the resulting DL-based tool demonstrated promising performance in predicting the T stage at the patient level, with accuracy comparable to that of expert gastroenterologists. We also evaluated the model’s ability to segment mesorectal lymph nodes. Although the tool may support preliminary screening for lymphadenopathies, the spatial alignment metrics indicate that further refinement is needed for reliable lymph node segmentation.

The good accuracy of the DL tool in distinguishing Tis and T1 from T2/T3 stages could provide substantial support for clinical decision-making, particularly when selecting between endoscopic resection and surgical approaches. By providing automated and reliable lesion segmentation and staging predictions, the DL tool has the potential to assist less experienced operators in interpreting R-EUS images, thereby reducing inter-operator variability and increasing confidence in staging. Furthermore, improvements in the accuracy of the DL model could facilitate real-time intraprocedural decision-making, guiding the choice between endoscopic submucosal dissection and surgical intervention, with or without adjuvant therapy, capitalizing on the ability of R-EUS to differentiate between mucosal, submucosal, and muscular layers—an area where rectal MRI has well-known limitations.

## Figures and Tables

**Table 1:** Baseline characteristics.

		**Patients’ Characteristics**
73 ± 9.5		Age
27 (54%)		Male
		**Tumors characteristics**
4.95 ± 2.37		Tumor length
8.94 ± 3.94		Distance from the anal verge
		T stage (standard of reference)
16 (32%)	Tis	
5 (10%)	T1	
17 (34%)	T2	
12 (24%)	T3	
		N stage (standard of reference)
38 (76%)	N0	
12 (24%)	N+	

T: Tumor; N: Lymph node.

**Table 2:** Predictive ability/diagnostic performance of the DL-tool in the dichotomization between Tis, T1 vs. T2, T3 for the training, validation and test sets.

F1-Score	Recall	Precision	Accuracy	Set
0.80	0.68	0.98	0.73	Training Set
0.80	0.68	0.90	0.70	Validation Set
0.75	0.77	0.73	0.74	Test Set

**Table 3:** Predictive ability/diagnostic performance of the DL-tool in the dichotomization between Tis vs. T1, T2, T3 for the training, validation and test sets.

F1-Score	Recall	Precision	Accuracy	Set
0.93	0.89	0.97	0.90	Training Set
0.88	0.88	0.87	0.80	Validation Set
0.86	0.89	0.83	0.80	Test Set

**Table 4:** Diagnostic performance of the DL-tool in T-staging of rectal cancer (results from the AUC-ROC analysis).

95% CI	AUC	Diagnostic Accuracy (%)	Tumor Stage
0.72–0.94	0.85	89.8	Tis
0.51–0.79	0.66	85.7	T1
0.79–0.97	0.91	89.8	T2
0.78–0.97	0.90	93.9	T3
0.85–0.99	0.95	95.92	Tis + T1

## Data Availability

The data presented in this study are available from the corresponding author upon reasonable request due to privacy restrictions.

## Abbreviations

The following abbreviations are used in this manuscript:

R-EUS	endoscopic ultrasound
TRUS	transrectal ultrasound
DL	deep learning
DSC	Dice Similarity Coefficient
MRI	magnetic resonance imaging
CAD	Computer-aided detection
AI	artificial intelligence
FPN	Feature Pyramid Network
ROC	Receiver Operating Characteristic
AUC	area under the curve
ESD	endoscopic submucosal dissection
ROIs	regions of interest
